# Donor-derived CD19-targeted chimeric antigen receptor T cells in adult transplant recipients with relapsed/refractory acute lymphoblastic leukemia

**DOI:** 10.1038/s41408-023-00881-z

**Published:** 2023-07-12

**Authors:** Ibrahim Aldoss, Samer K. Khaled, Yan Wang, Xiuli Wang, Joycelynne Palmer, Mary C. Clark, Jamie R. Wagner, Jinny Paul, Vibhuti Vyas, Christine E. Brown, Stephen J. Forman

**Affiliations:** 1 Hematological Malignancies Research Institute, City of Hope, Duarte, California USA; 2 Gehr Family Center for Leukemia Research, City of Hope, Duarte, California USA; 3 Department of Hematology/Hematopoietic Cell Transplantation, City of Hope, Duarte, California USA; 4 Department of Computational and Quantitative Sciences, Division of Biostatistics, Beckman Research Institute, City of Hope, Duarte, California USA; 5 T Cell Therapeutics Research Laboratories, City of Hope, Duarte, California USA; 6 Department of Clinical and Translational Project Development, City of Hope, Duarte, California USA

**Keywords:** Translational research, Cancer immunotherapy

Dear Editor,

CD19-targeted chimeric antigen receptor (CAR) T cell therapy induces remission in the majority of patients with relapsed and refractory (r/r) B-cell acute lymphoblastic leukemia (ALL), including those who have exhausted all other available salvage therapies [1–3]. While this novel therapy has curative potential, commercially available CAR T cell products are approved for autologous use only and require the collection of a patient’s own T cells, which is an obstacle for some patients with advanced r/r ALL. Successful preparation and infusion of autologous CAR T cells requires a balance of salvage therapy that allows for an adequate number of T cells in the peripheral blood to generate an autologous product as well as disease stabilization during the CAR T cell manufacture period. This represents a challenge in patients with relapsed proliferative ALL, for whom chemotherapy only temporarily induces bone marrow aplasia and cytopenia, but upon recovery, white blood cells are predominantly blasts. To overcome this hurdle, efforts are ongoing to optimize novel off-the-shelf CAR T cell platforms that use healthy donor cells and are ready to administer on an as-needed basis [4, 5].

Recipients of allogeneic hematopoietic cell transplantation (alloHCT) who experience subsequent ALL relapse represent a unique population to study the use of donor-derived CAR T cell products; this population has potentially a ready source of donor-derived CAR T cells as long as their original donors are willing to donate additional T cells akin to donor lymphocyte infusion (DLI). Brudno and colleagues investigated the use of T cells collected from prior donors for recipients of alloHCT who subsequently experienced a relapse of their B-cell malignancy [6]. Twenty patients, including five with r/r ALL, received donor-derived CAR T cells; in this study, patients did not receive lymphodepletion (LD), and most patients (80%) had a DLI from the same donor before CAR T cell infusion. This approach was feasible and safe and led to complete remission (CR)/CR with incomplete count recovery (CRi) in four of five patients with r/r ALL [6]. Considering the challenge of collecting autologous T cells in the setting of proliferative relapsed ALL, the use of donor T cells for CAR T cell manufacturing represents an attractive opportunity to expand the use of CAR T cell therapy in ALL following post-alloHCT relapse.

We recently published the results of a phase 1/2 prospective study assessing memory-enriched CD19CAR T cell therapy in adults with r/r B-ALL (NCT02146924; City of Hope Institutional Review Board #13447) [3]. The study enrolled 58 patients, including 37 (64%) who had relapsed after alloHCT. The study permitted donor leukapheresis in prior recipients of alloHCT if the donor was a sibling, regardless of the degree of HLA compatibility. Here, we performed secondary analyses of patients who had prior alloHCT (*n* = 37) and compared the characteristics and outcomes of patients who had donor (*n* = 12) versus autologous (*n* = 25) CAR T cell products. All enrolled patients and their collected donors signed informed consent. Patient characteristics were summarized using descriptive statistics, including median (range) for continuous variables and count (percentage) for categorical variables. Clopper–Pearson exact method was used to construct the 95% confidence intervals (CI) for CR/CRi rate. Overall survival (OS) was estimated using the Kaplan–Meier method, and the log-log transformation method with Greenwood’s formula for standard error was used to construct 95% CIs of OS. All statistical analyses were done in R version 4.2.0 (R Foundation, Vienna, Austria).

Of the 58 adults with r/r ALL enrolled in the study, 46 (79%) received CAR T cell infusion at the recommended phase 2 dose (RP2D) of 200 × 10^6^ CAR+ T cells. Of the 37 patients who had relapsed post alloHCT, 12 (32%) had T cells collected directly from their sibling donors after enrollment, and 25 (68%) underwent autologous leukapheresis. Refer to Supplementary Fig. 1 for the consort diagram and Supplementary Table 1 for characteristics of enrolled patients by status of prior alloHCT. Median donor CD3 chimerism at the time of enrollment was 98.7% (range, 79.99–100%) and 100% (range, 91.3–100%) for donor-derived and autologous cohorts, respectively. Patients who used donor leukapheresis products compared to patients who underwent autologous leukapheresis tended to be younger (median age: 30 vs. 42 years), had higher median marrow blasts at enrollment (63% vs. 0%) and pre-LD (70% vs. 0%), more frequently had poor-risk cytogenetics (67% vs. 24%), were less likely to have extramedullary disease at enrollment (30% vs. 60%) and pre-LD (14% vs. 43%), and their prior transplant donor was more likely to be a sibling (100% vs. 48%). Of the 12 sibling donors who underwent leukapheresis, seven were fully matched, two were mismatched (both were 9/10 HLA matched), and three were haploidentical. Only 6 (50%) and 23 (92%) who used donor leukapheresis products and who underwent autologous leukapheresis were eventually infused with CAR T cells at RP2D, respectively (Supplementary Table 2). Table 1 illustrates the characteristics of the 37 patients who were enrolled after post-alloHCT relapse.

**Table 1:** Patient characteristics by T cell source.

	Median (range) or *n* (%)		
Variables	Overall, *N* = 37	T-cell collection	
		Donor, *N* = 12	Autologous, *N* = 25
Age at infusion (years)	36 (26, 70)	30 (27, 36)	42 (26, 70)
Not applicable^a^	8	6	2
Sex			
Female	17 (46)	5 (42)	12 (48)
Male	20 (54)	7 (58)	13 (52)
Race/ethnicity			
Asian, Non-Hispanic or Latino	3 (8.1)	1 (8.3)	2 (8.0)
Black, Non-Hispanic or Latino	1 (2.7)	0 (0)	1 (4.0)
Caucasian, Hispanic or Latino	19 (51)	6 (50)	13 (52)
Caucasian, Non-Hispanic or Latino	12 (32)	4 (33)	8 (32)
Unknown, Hispanic or Latino	2 (5.4)	1 (8.3)	1 (4.0)
BM Blasts ≥ 5% and/or EMD at enrollment	32 (86)	11 (92)	21 (84)
BM Blasts % at enrollment	2 (0, 100)	63 (0, 95)	0 (0, 100)
EMD at enrollment			
Yes	19 (51)	4 (33)	15 (60)
*CNS/orbit involved*	*8 (21.6)*	*2 (16.7)*	*6 (24.0)*
*Non-CNS EMD*	*11 (29.7)*	*2 (16.7)*	*9 (36.0)*
No	18 (49)	8 (67)	10 (40)
Cytogenetic risk at diagnosis			
Good risk	5 (14)	2 (17)	3 (12)
Standard risk	15 (41)	2 (17)	13 (52)
Poor risk	14 (38)	8 (67)	6 (24)
Undetermined	3 (8.1)	0 (0)	3 (12)
Philadelphia chromosome-positive	6 (16)	3 (25)	3 (12)
Philadelphia chromosome-like	15 (41)	5 (42)	10 (40)
KMT2A-rearrangement	4 (11)	2 (17)	2 (8)
Total number of all drug regimens	4 (1, 9)	4 (1, 7)	3 (1, 9)
Prior blinatumomab	23 (62)	8 (67)	15 (60)
Prior inotuzumab	11 (30)	1 (8.3)	10 (40)
Prior AlloHCT donor type			
Related (non-sibling)	2 (5.4)	0 (0)	2 (8.0)
Sibling	24 (65)	12 (100)	12 (48)
Umbilical cord	1 (2.7)	0 (0)	1 (4.0)
Unrelated	10 (27)	0 (0)	10 (40)
Prior AlloHCT HLA match degree			
Haploidentical	12 (32)	3 (25)	9 (36)
HLA Identical Sibling	12 (32)	7 (58)	5 (20)
HLA matched, unrelated	3 (8.1)	0 (0)	3 (12)
HLA mismatched sibling	2 (5.4)	2 (17)	0 (0)
HLA mismatched, unrelated	7 (19)	0 (0)	7 (28)
Unrelated umbilical cord	1 (2.7)	0 (0)	1 (4.0)
BM blasts ≥ 5% and/or EMD at LD			
Yes	24 (75)	7 (78)	17 (74)
No	8 (25)	2 (22)	6 (26)
Missing	5	3	2
BM blasts % at LD	4 (0, 92)	70 (0, 91)	0 (0, 92)
Missing	5	3	2
EMD at LD			
Yes	11 (37)	1 (14)	10 (43)
No	19 (63)	6 (86)	13 (57)
Missing	7	5	2
Received post leukapheresis cytoreduction therapy	26 (70)	5 (42)	21 (84)
Received 200 × 10^6^ CAR T cells	29 (78)	6 (50)	23 (92)

^a^Did not proceed to infusion.

*CNS* central nervous system, *EMD* extramedullary disease, *BM* bone marrow, *LD* lymphodepletion.

CAR T cell products generated from donor versus autologous cells were overall comparable in terms of median manufacturing time (12 vs. 12 days), viability (89.5% vs. 90.1%), and fold expansion (14.9 and 18.0), respectively. Following infusion, grade ≥ 3 toxicities at least possibly attributed to CAR T cell therapy were comparable for donor-derived and autologous cohorts (Supplementary Table 3). We observed any grade/grade ≥3 cytokine release syndrome (CRS) per Lee et al. criteria [7] in 100%/17 and 91%/4% in donor-derived and autologous cohorts, and any grade/grade ≥3 neurotoxicity in 100%/33 and 70%/4%, respectively (Supplementary Table 4). A single patient in the autologous cohort who had received a prior haploidentical transplant developed moderate chronic skin and liver graft-versus-host disease (GVHD) with an onset of 73 days after CAR T cell infusion; GVHD was controlled with steroids and tacrolimus. The CR/CRi rate for patients who received donor-derived and autologous CAR T cell products and who were evaluable for response was 83% (95%CI: 35.9–99.6%) and 95% (95%CI: 77.2–99.9%), respectively, with only one patient in each cohort who was refractory for treatment. The refractory patient who received a donor-derived product had *KMT2A* rearrangement and experienced early marrow and extramedullary CD19-negative disease progression shortly after T cells infusion. Post response, 3 (60%) and 7 (33%) patients who received donor-derived and autologous products, respectively, underwent a second alloHCT.

With a median follow-up of 9.4 months (range, 0.6–58.4), the median OS for all patients who received CAR T cell infusion was 11.6 months (95%CI: 8.7–not reached). By cohort, the median OS was not reached and 11 months (95%CI: 5.3–not reached) for patients who received donor-derived products and autologous products, respectively (Fig. 1A). Cause of death for patients who were evaluable for response are shown in Supplementary Table 5. Post infusion CAR T cell in vivo expansion and persistence were overall comparable for donor-derived and autologous products as measured by a quantitative PCR assay to detect the WPRE transgene within the lentiviral vector (Fig. 1B, C).

**Fig. 1:**
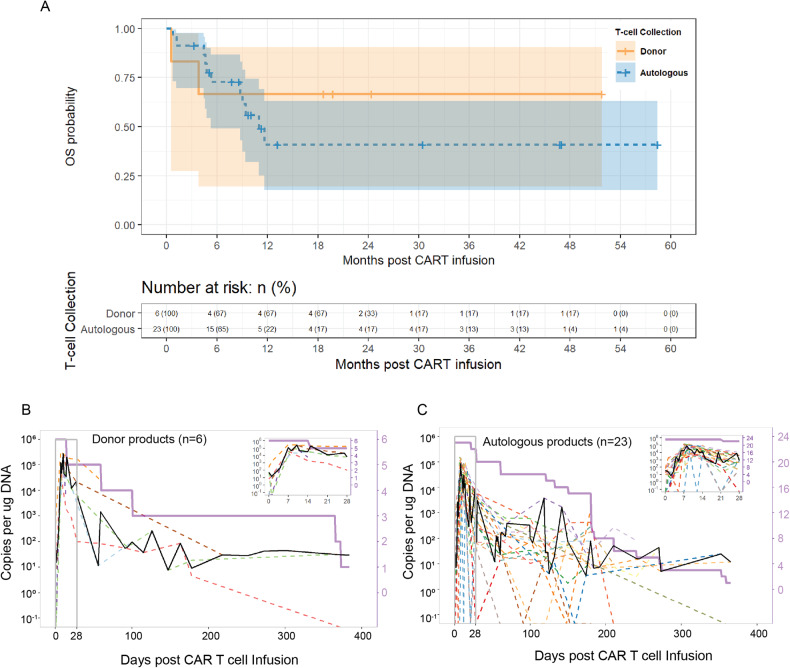
Outcomes of patients who received autologous or donor-derived CAR T cell products. **A** Kaplan–Meier survival curves for relapsed patients after allogeneic HCT who were infused with memory-enriched CD19CAR T cells at RP2D. The orange curve represents patients who received donor-derived CAR T cells, and the blue curve represents patients who received autologous CAR T cells. **B** Post infusion in vivo expansion and persistence in patients who received donor-derived CAR T cells. **C** Post infusion in vivo expansion and persistence in patients who received autologous CAR T cells.

Overall, we determined that using donor-derived products was feasible and potentially allowed us to overcome one current limitation of commercially approved CAR T cell products in patients with r/r ALL with proliferative disease. Despite the high burden of marrow disease at enrollment and pre-LD in the cohort who received donor-derived products, all patients achieved CR/CRi except for one patient with *KMT2A* rearrangement who progressed during the first week with CD19-negative disease. Nonetheless, the dropout rate of patients who had donor-collected cells remained high at 50%, which reflects the severity of illness in these patients at the time of relapse, including high disease burden and susceptibility to various complications. We did not observe unexpected toxicities in patients who received donor-derived CAR T cells, which was anticipated considering that donor CD3 chimerism was high in most patients at the time of enrollment. Moreover, we showed that OS did not appear to be inferior to that of patients who received autologous CAR T cell products and second alloHCT was feasible post response to CAR T cells.

In contrast to the experience of Brudno et al. [6], we only included adults with r/r ALL and administered a standard LD regimen prior to CAR T cell infusion. Furthermore, we restricted our donor cohort to patients with sibling donors, although we extended inclusion to haploidentical and mismatched donors, which is highly relevant considering the increasing use of haploidentical donors for alloHCT. Additionally, while most patients (80%) had prior DLI in the Brudno et al. study, none of our patients received DLI before donor-derived CAR T cell infusion, which implies that prior DLI is not a key step for a safe donor-derived CAR T cell preparation.

Our study is limited by the small number of patients who used donor-derived products, which precludes extensive analyses and robust conclusions. Nonetheless, our report provides the proof-of-concept of the feasibility and safety of donor T cells for CAR T cell production in recipients of prior alloHCT who experience relapse. A larger study confirming our observation using commercially available CAR T cell products (e.g., brexucabtagene autoleucel, tisagenlecleucel) in ALL will be of great value and could extend the use of CAR T cells in r/r disease.

## Supplementary information

Supplemental Data

## Data Availability

The datasets generated during and/or analyzed during the current study are available from the corresponding author upon reasonable request.
