# Supplementary material for: Donor-derived CD19-targeted chimeric antigen receptor T cells in adult transplant recipients with relapsed/refractory acute lymphoblastic leukemia
Source: Blood Cancer J. 2023 Jul 12;13(1):107. doi: 10.1038/s41408-023-00881-z (PMC10338536; doi:10.1038/s41408-023-00881-z)
Supplement: Supplementary file 1 — Supplemental Data [file 41408_2023_881_MOESM1_ESM.pdf]

Supplemental Figure 1

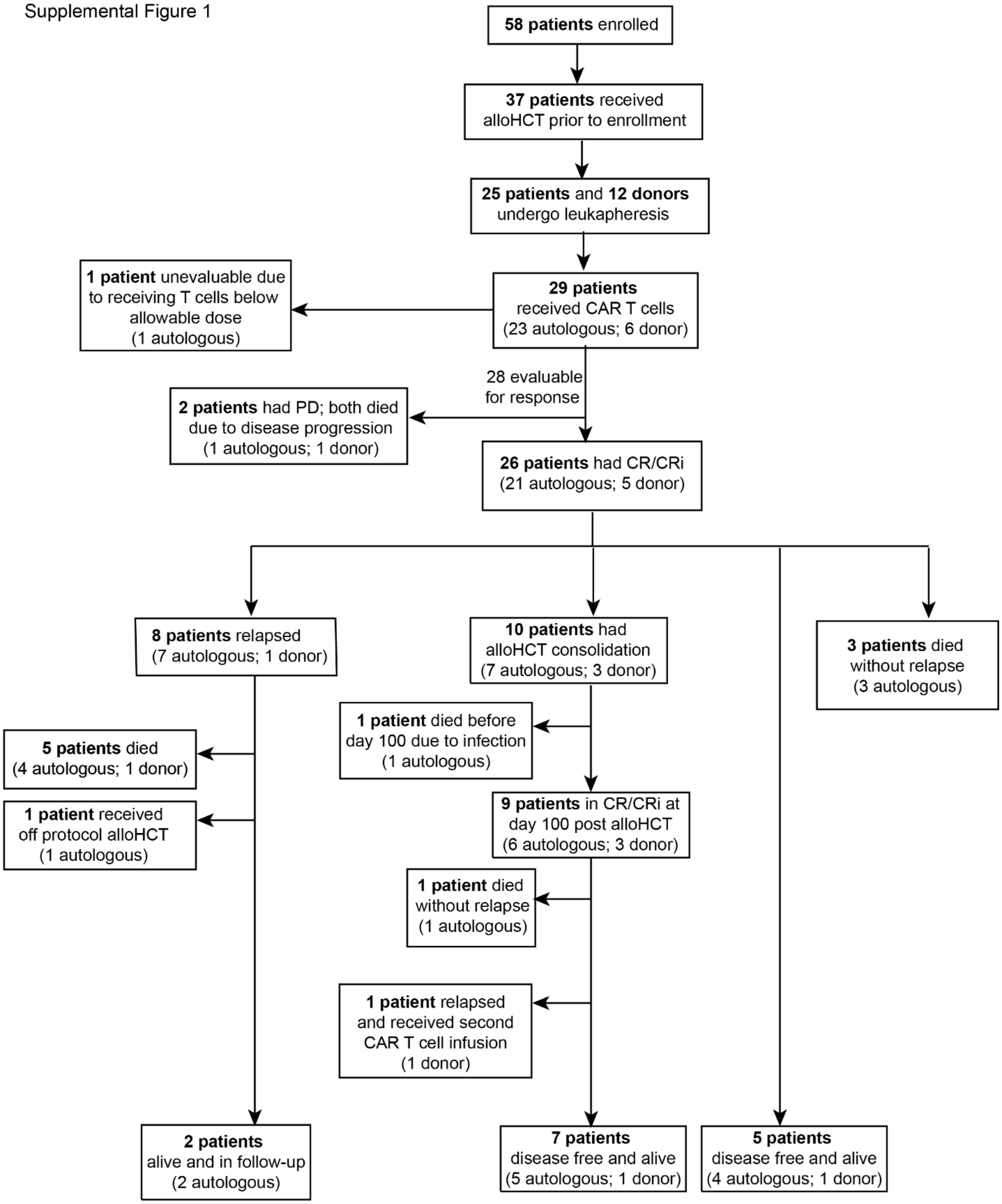

Supplemental Figure 1. Consort diagram of patients who received alloHCT prior to CD19-CAR T cells. The outcome of patients (n=37) who received 200×10^6 CD19-CAR T cells is depicted. Patients who received autologous and donor-derived (donor) products are indicated.

Supplemental Table 1. Patient characteristics by prior AlloHCT

Variables	Median (range) or n (%)		
	Overall, N=58	Received alloHCT prior to study enrollment	
		No, N=21	Yes, N = 37
**Age at infusion (yrs)**	38 (22, 72)	43 (22, 72)	36 (26, 70)
Not applicable*	12	4	8
**Sex**			
Female	24 (41)	7 (33)	17 (46)
Male	34 (59)	14 (67)	20 (54)
**Race/Ethnicity**			
Asian, Non-Hispanic or Latino	3 (5.2)	0 (0)	3 (8.1)
Black, Non-Hispanic or Latino	1 (1.7)	0 (0)	1 (2.7)
Caucasian, Hispanic or Latino	29 (50)	10 (48)	19 (51)
Caucasian, Non-Hispanic or Latino	21 (36)	9 (43)	12 (32)
Pacific Islander and Asian, Non-Hispanic or Latino	1 (1.7)	1 (4.8)	0 (0)
Unknown, Hispanic or Latino	3 (5.2)	1 (4.8)	2 (5.4)
**T-cell collection**			
Donor	12 (21)	0 (0)	12 (32)
Patient	46 (79)	21 (100)	25 (68)
**BM Blasts ≥5% and/or EMD at enrollment**			
Yes	51 (88)	19 (90)	32 (86)
No	7 (12)	2 (9.5)	5 (14)
**BM Blasts % at enrollment**	30 (0, 100)	40 (0, 97)	2 (0, 100)
**EMD at enrollment**	28 (48)	9 (43)	19 (51)
**Cytogenetic risk at diagnosis**			
Good risk	8 (14)	3 (14)	5 (14)
Standard risk	21 (36)	6 (29)	15 (41)
Poor risk	23 (40)	9 (43)	14 (38)
Undetermined	6 (10)	3 (14)	3 (8.1)
**Total number of all drug regimens**	4 (1, 9)	3 (1, 5)	4 (1, 9)
**Prior blinatumomab use**	38 (66)	15 (71)	23 (62)
**Prior inotuzumab**	17 (29)	6 (29)	11 (30)
**BM Blasts ≥5% and/or EMD at LD**			
Yes	41 (82)	17 (94)	24 (75)
No	9 (18)	1 (5.6)	8 (25)
Missing	8	3	5
**BM Blasts % at LD**	18 (0, 95)	45 (0, 95)	4 (0, 92)
Missing	8	3	5
**EMD at LD**			
Yes	16 (34)	5 (29)	11 (37)
No	31 (66)	12 (71)	19 (63)
Missing	11	4	7

*Did not proceed to infusion

Supplemental Table 2. Characteristics of patients who received CAR T cell products by T cell source

	Median (range) or n (%)		
		T-cell collection	
**Variables**	**Overall, N = 29**	**Donor, N = 6**	**Patient, N = 23**
**Age at infusion (yrs)**	36 (26, 70)	30 (27, 36)	42 (26, 70)
**Sex**			
Female	14 (48)	3 (50)	11 (48)
Male	15 (52)	3 (50)	12 (52)
**Race/Ethnicity**			
Asian, Non-Hispanic or Latino	3 (10)	1 (17)	2 (8.7)
Black, Non-Hispanic or Latino	1 (3.4)	0 (0)	1 (4.3)
Caucasian, Hispanic or Latino	14 (48)	3 (50)	11 (48)
Caucasian, Non-Hispanic or Latino	9 (31)	1 (17)	8 (35)
Unknown, Hispanic or Latino	2 (6.9)	1 (17)	1 (4.3)
**BM Blasts ≥ 5% and/or EMD at enrollment**	24 (83)	5 (83)	19 (83)
**BM Blasts % at enrollment**	1 (0, 100)	40 (0, 95)	0 (0, 100)
**EMD at enrollment**			
Yes	16 (55)	2 (33)	14 (61)
CNS/orbit involved	*6 (21)*	*1 (16.7)*	*5 (22)*
Non-CNS EMD	*10 (34)*	*1 (16.7)*	*9 (39)*
No	13 (45)	4 (67)	9 (39)
**Cytogenetic risk at diagnosis**			
Good risk	5 (17)	2 (33)	3 (13)
Standard risk	11 (38)	0 (0)	11 (48)
Poor risk	10 (34)	4 (67)	6 (26)
Undetermined	3 (10)	0 (0)	3 (13)
**Philadelphia chromosome-positive**	5 (17)	2 (33)	3 (13)
**Philadelphia chromosome-like**	12 (41)	3 (50)	9 (39)
**KMT2A-rearrangement**	3 (10)	1 (17)	2 (9)
**Total number of all drug regimens**	3 (1, 9)	4 (1, 6)	3 (1, 9)
**Prior blinatumomab**	17 (59)	4 (67)	13 (57)
**Prior inotuzumab**	11 (38)	1 (17)	10 (43)
**Prior AlloHCT Donor Type**			
Related (non-sibling)	2 (6.9)	0 (0)	2 (8.7)
Sibling	17 (59)	6 (100)	11 (48)
Umbilical cord	1 (3.4)	0 (0)	1 (4.3)
Unrelated	9 (31)	0 (0)	9 (39)
**Prior AlloHCT HLA Match Degree**			
Haploidentical	11 (38)	3 (50)	8 (35)
HLA Identical Sibling	8 (28)	3 (50)	5 (22)
HLA matched, Unrelated	3 (10)	0 (0)	3 (13)
HLA Mismatched, unrelated	6 (21)	0 (0)	6 (26)
Unrelated umbilical cord	1 (3.4)	0 (0)	1 (4.3)
**BM Blasts ≥ 5% and/or EMD at LD**	21 (72)	4 (67)	17 (74)
**BM Blasts % at LD**	1 (0, 92)	42 (0, 91)	0 (0, 92)
**EMD at LD**	11 (38)	1 (17)	10 (43)
**Received post leukapheresis cytoreduction therapy**	26 (90)	5 (83)	21 (91)

Supplemental Table 3. Grade 3 or Higher Adverse Events at Least Possibly Attributed to CAR T cell Infusion Through Day 28

Adverse Event	Donor collected T cells (%) [n= 6]	Autologous T cells (%) [n= 23]
Febrile neutropenia	5 (83.3)	15 (65.2)
Fibrinogen decreased	3 (50)	1 (4.3)
Lymphocyte count decreased	3 (50)	7 (30.4)
Platelet count decreased	0	5 (21.7)
Anemia	0	4 (17.4)
Hypoxia	3 (50)	4 (17.4)
Nausea	1 (16.7)	1 (4.3)
Vomiting	1 (16.7)	0
Fever	1 (16.7)	1 (4.3)
Pain	1 (16.7)	0
Cytokine Release Syndrome (CRS)	1 (16.7)	1 (4.3)
Blood bilirubin increased	1 (16.7)	1 (4.3)
Neutrophil count decreased	1 (16.7)	3 (13)
Weight gain	1 (16.7)	0
White blood cell decreased	1 (16.7)	3 (13)
Hypertriglyceridemia	1 (16.7)	0
Tumor lysis syndrome	1 (16.7)	0
Back pain	1 (16.7)	0
Pain in extremity	1 (16.7)	0
Dysphasia	1 (16.7)	0
Encephalopathy	1 (16.7)	1 (4.3)
Headache	1 (16.7)	0
Somnolence	1 (16.7)	0
Hypotension	1 (16.7)	3 (13)
Abdominal pain	0	1 (4.3)
Fatigue	0	1 (4.3)
Hepatic failure	0	1 (4.3)
Alanine aminotransferase increased	0	1 (4.3)
Aspartate aminotransferase increased	0	1 (4.3)
Anorexia	0	1 (4.3)
Hypokalemia	0	1 (4.3)
Hypophosphatemia	0	1 (4.3)
Acute kidney injury	0	1 (4.3)
Hematuria	0	1 (4.3)
Hypertension	0	1 (4.3)

Supplemental Table 4. Neurotoxicity and cytokine release syndrome at least possibly attributed to CAR T cell therapy

Adverse Event*	T cell Collection								Overall, n=29			
	Donor, n=6				Autologous, n=23							
	Gr1	Gr2	Gr3	Total (%)	Gr1	Gr2	Gr3	Total (%)	Gr1	Gr2	Gr3	Total (%)
**Neurotoxicity**												
Headache	2	2	1	5 (83.3)	7	2		9 (39.1)	9	4	1	14 (48.3)
Dizziness	3			3 (50)	6			6 (26.1)	9			9 (31)
Confusion	3	1		4 (66.7)	4			4 (17.4)	7	1		8 (27.6)
Lethargy	3			3 (50)	2	2		4 (17.4)	5	2		7 (24.1)
Tremor	2			2 (33.3)	2			2 (8.7)	4			4 (13.8)
Somnolence			1	1 (16.7)	1	1		2 (8.7)	1	1	1	3 (10.3)
Dysphasia			1	1 (16.7)	2			2 (8.7)	2		1	3 (10.3)
Memory impairment	1			1 (16.7)	2			2 (8.7)	3			3 (10.3)
Peripheral sensory neuropathy				0 (0)	3			3 (13)	3			3 (10.3)
Encephalopathy			1	1 (16.7)			1	1 (4.3)			2	2 (6.9)
Cognitive disturbance				0 (0)	2			2 (8.7)	2			2 (6.9)
Presyncope				0 (0)		1		1 (4.3)		1		1 (3.4)
Dysarthria	1			1 (16.7)				0 (0)	1			1 (3.4)
**Max grade of any neurotoxicity per patient**	2	2	2 (33.3)	6 (100)	12	3	1 (4.3)	16 (69.6)	14	5	3 (10.3)	22 (75.9)
**Max grade of CRS per patient**	1	4	1 (16.7)	6 (100)	6	14	1 (4.3)	21 (91.3)	7	18	2 (6.9)	27 (93.1)

* Toxicity was assessed using CTCAE v4.03. CRS grade was defined using Lee et al. criteria.

Supplemental Table 5. Cause of Death among response-evaluable patients

Cause of Death	T-cell collection*	
	Donor, N = 6	Autologous, N = 22
Disease progression	2	4
Infection	0	4
Multi-organ failure due to sepsis	0	1
Respiratory failure	0	2
Alive at last contact or censoring	4	11

*: One additional patient with autologous T cell collection was unevaluable for response due to receiving lower than allowable infusion dose due to low manufacturing yield. This patient expired at day 36 of widespread sepsis.
